# Heat responsive gene *StGATA2* functions in plant growth, photosynthesis and antioxidant defense under heat stress conditions

**DOI:** 10.3389/fpls.2023.1227526

**Published:** 2023-07-11

**Authors:** Xi Zhu, Huimin Duan, Hui Jin, Shu Chen, Zhuo Chen, Shunwei Shao, Jinghua Tang, Yu Zhang

**Affiliations:** ^1^ Key Laboratory of Tropical Fruit Biology, Ministry of Agriculture and Rural Affairs of China, Zhanjiang, China; ^2^ Key Laboratory of Hainan Province for Postharvest Physiology and Technology of Tropical Horticultural Products, South Subtropical Crops Research Institute, Chinese Academy of Tropical Agricultural Sciences, Zhanjiang, China; ^3^ National Key Laboratory for Tropical Crop Breeding, Sanya Research Institute, Chinese Academy of Tropical Agricultural Sciences, Sanya, China; ^4^ College of Horticulture and Forestry Science, Huazhong Agricultural University, Wuhan, China

**Keywords:** potato, heat stress, StGATA2, morphological phenotypes, photosynthesis, transpiration

## Abstract

**Backgrounds:**

Potato is sensitive to heat stress particularly during plant seedling growth. However, limited studies have characterized the expression pattern of the *StGATA* family genes under heat stress and lacked validation of its function in potato plants.

**Methods:**

Potato plants were cultivated at 30°C and 35°C to induce heat stress responses. qRT-PCR was carried out to characterize the expression pattern of *StGATA* family genes in potato plants subjected to heat stress. *StGATA2* loss-of-function and gain-of-function plants were established. Morphological phenotypes and growth were indicated by plant height and mass. Photosynthesis and transpiration were suggested by stomatal aperture, net photosynthetic rate, transpiration rate, and stomatal conductance. Biochemical and genetic responses were indicated by enzyme activity and mRNA expression of genes encoding CAT, SOD, and POD, and contents of H_2_O_2_, MDA, and proline.

**Results:**

The expression patterns of *StGATA* family genes were altered in response to heat stress. StGATA2 protein located in the nucleus. *StGATA2* is implicated in regulating plant height and weight of potato plants in response to heat stresses, especially acute heat stress. *StGATA2* over-expression promoted photosynthesis while inhibited transpiration under heat stress. *StGATA2* overexpression induced biochemical responses of potato plant against heat stress by regulating the contents of H_2_O_2_, MDA and proline and the activity of CAT, SOD and POD. *StGATA2* overexpression caused genetic responses (CAT, SOD and POD) of potato plant against heat stress.

**Conclusion:**

Our data indicated that *StGATA2* could enhance the ability of potato plants to resist heat stress-induced damages, which may provide an effective strategy to engineer potato plants for better adaptability to adverse heat stress conditions.

## Highlights

1. Expression patterns of *StGATAs* are altered in response to heat stresses;
2. *StGATA2* affects plant morphological phenotypes and growth under heat stress conditions;
3. The photosynthesis and transpiration are regulated by *StGATA2* in response to heat stress;
4. *StGATA2* induces biochemical and genetic responses of potato plants to heat stress.

## Introduction

The GATA-binding transcription factors comprise a protein family whose members contain either one or two highly conserved zinc finger DNA-binding domains (Teakle et al., 2002).GATA factors normally recognize the consensus sequence W-GATA-R (W, thymidine (T) or an adenosine (A); R, guanidine (G) or adenosine (A)) (Behringer and Schwechheimer, 2015). The DNA binding domain of GATA factors is constituted by a type IV zinc finger in the form CX_2_CX_17-20_CX_2_C followed by a highly basic region (Gronenborn, 2005). GATA proteins present two of zinc fingers, where only the C-terminal finger (C-finger) is involved in DNA binding, and the N-terminal zinc finger (N-finger) can modulate the binding of the C-finger to specific GATA sites, bind DNA with different specificity, or mediate the interaction with transcription cofactors of the friend of GATA family (Bates et al., 2008). The majority of the plant GATA factors contain a single zinc finger domain and mostly fall into two different categories: those with 17-residue loops (CX_2_CX_17_CX_2_C; also called zinger finger type IVa) and those with 18-residue loops (CX_2_CX_18_CX_2_C; also called zinger finger type IVb) (Scazzocchio, 2000).

Members of GATA family have been identified in plant organisms. Reyes et al. documented the existence of 29 different loci encoding putative GATA factors in Arabidopsis and 28 in rice (Reyes et al., 2004). In rapeseed, 96 *GATAs* have been identified and classified into 4 subfamilies based on the phylogenetic relationships, DNA binding domains and intron-exon structures (Zhu et al., 2020). Unevenly distributing on 21 chromosomes, 79 *GATAs* have been characterized in wheat (Feng et al., 2022a). In *Ophiorrhiza pumila* (Rubiaceae), genome-wide survey identified a total of 18 *GATA* genes classified into 4 subfamilies (Shi et al., 2022). In *Brachypodium distachyon*, Peng et al. systematically identified 28 GATA transcription factors distributing on 5 chromosomes (Peng et al., 2021). As for *Solanum* (Family: *Solanaceae*) plants, 30 *GATAs* were recognized in tomato (*Solanum lycopersicum*) by genome-wide analysis (Yuan et al., 2018). Recently, Yu et al. confirmed 49 *GATAs* randomly distributing on 12 chromosomes in potato (*Solanum tuberosum*) (Yu et al., 2022).

Generally speaking, there appears to be evolutionary conservation with regard to function of GATA factors, which has been validated to play roles In the control of normal physiological processes, such as chloroplast development, plant architecture (Hudson et al., 2013), chlorophyll synthesis, glucose metabolism (Bi et al., 2005), apical meristem and flower development (Zhao et al., 2004). Additionally, *GATAs* are light-regulated genes and respond to signals from the circadian clock (Manfield et al., 2007). Studies have revealed the transcript abundance of several *GATAs* is responsive to salinity, drought, exogenous ABA, and gibberellins (Gupta et al., 2017; Guo et al., 2021). However, there is limited studies on the responses of *GATAs* to heat stresses in potato plants. Herein, the current study described the expression patterns of *StGATAs* under heat stress conditions. Further, we analyzed the functional aspects of *StGATA2* and stress responses-related genes. The biological functions of *GATA2* gene have been reported in biological processes, such as photomorphogenesis (Luo et al., 2010). However, its responses to abiotic or biotic stresses are rarely reported.

## Materials and methods

### Plant material and heat stress

Potato (*Solanum tuberosum* L.) cultivar “Atlantic” was used in this study, which is mainly due to its high yields of tubers and its resistance to common scab, latent and mild mosaics. Potato plants were *in vitro* planted in Murashige and Skoog (MS) medium with pH adjusted among 5.8-6.0. The medium was supplemented with 3% sucrose for seedling growth. To induce tuber generation, 8% sucrose was utilized. The cultivation was conducted in a controlled biotron, with the following conditions: 22°C/15°C (day/night), 16-h/8-h light/dark photoperiod (2,800 Lux), and 50% air humidity. Potato tubers with a sprouted bud of 1 mm in height were transferred into soil and vermiculite (1:1, v/v) pots measuring 18 cm × 26 cm × 27 cm and maintained for 5 weeks. To induce heat stress, the plants were next fostered under mild (30°C) and acute (35°C) conditions. Potato leaves were collected for detecting transcriptional patterns of *StGATA* family genes, 0 h, 1 h, 2 h, 4 h, 8 h, 12 h, 24 h, and 48 h after heat treatment. For the quantitation of *StGATAs* genes expression, there were 144 seedlings (1 line × 2 heat treatments × 8 different time periods × 3 replications × 3 pots); For the quantitation of heat-responsive genes, there were 630 seedlings (7 lines × 2 heat treatments × 5 different time periods × 3 replications × 3 pots); For the examination of physiological and photosynthetic indexes, there were 630 seedlings (7 lines × 2 heat treatments × 5 different time periods × 3 replications × 3 pots).

### Construction of transgenic plants

To generate potato plants highly expressing *StGATA2* gene (OE for short), StGATA2 protein-encoding gene (GenBank Accession No. XM_006347854.2) was amplified using the specific primers (forward, 5’-CTCGAGATGGATGTCTACGGCGTGCACTCT-3’ and reverse 5’-GTCGACGCAGACCCGAAAGTGATGTCCGTACATTC-3’) (Bioeditas, Shaanxi, China). The PCR products were cloned into pBI121-EGFP plasmid according to a previous method (Li et al., 2020). The constructed plasmid was introduced into *Agrobacterium tumefaciens* strains LBA4404, followed by infecting the potato tuber slide (2 mm) according to Si’s method (Si et al., 2003). Potato plants with *StGATA2* knocked down (Ri for short) were constructed with a previous method (Lu et al., 2019). The sense cDNA sequence was amplified using forward primer (Kpn I) and reverse primer (EcoR I) and inserted as an Kpn I-EcoR I fragment into pHANNIBAL (pHAN-StMAPK1-F); The anti-sense cDNA sequence was amplified using forward primer (Hind III) and reverse primer (BamH I), and inserted as a Hind III-BamH I fragment into pHANNIBAL (pHAN-StGATA2-R). The pHAN-StGATA2-RF were subcloned at Sac I and Spe I sites into pART vector (pART-StGATA2-RNAi). pART-StGATA2-RNAi was introduced into LBA4404, which was used to infect potato tuber slides.

After agrobacterium-mediated transformation, the tuber slides were cultivated on a solid medium in the dark at 28°C for 48 h. Then plants were continually cultivated on differentiation media in the dark at 22°C with 16-h/8-h light/dark photoperiod (2,400 Lux). The medium was changed every 12 days and the plants were cultured for about 3 to 4 weeks for shoot differentiation. Plant shoots were cut after growing to 1.5 cm and the shoots were transferred to the rooting medium for screening with 75% mg/mL of kanamycin. After 3 weeks of cultivation on MS medium, the leaves were collected for detecting *StGATA2* by PCR using the specific primers for RNAi (forward: 5’-ATGGATGTCTACGG CGTGCACTCT-3’ and reverse: 5’- ATTAGCCGGAAAATTACTAAATGAAT-3’) and NPT II (forward: 5’-CTCACCTTGCTCCTGCCGAGA-3’ and reverse: 5’-CGCCTTGAGCCTGG CGAACAG-3’).

### qRT-PCR

mRNA expression of *StGATA* family genes was examined 0 h, 1 h, 2 h, 4 h, 8 h, 12 h, 24 h, and 48 h after heat stress that has been described as abovementioned. For *StGATA2* mRNA expression in transgenic plants, the leaves were collected 3 weeks after cultivation on MS medium. To examine the expression of heat stress genes (*StSOD*, *StCAT*, *StPOD* and *StP5CS*), plants leaves were collected 0 h, 8 h, 12 h, 24 h and 48 h after heat treatments. TRIzol RNA Extraction Kit (Invitrogen, Carlsbad, CA, USA). The first-strand cDNA was synthesized using the First-Strand cDNA Synthesis Kit (TransGen Biotech, Beijing, China). qPCR was performed using 100 ng of cDNA, 10 μL of SYBR Premix Ex Taq (2 ×) (Takara, Tokyo, Japan), and 0.8 μL of specific primers (0.5 μM) on ABI3000 System (Applied Biosystems, Foster City, CA, USA). The reaction procedures were: one cycle at 94°C for 3 min and 36 cycles of amplification at 94°C for 45 s, 59°C for 34 s, and 72°C for 1 min. The relative mRNA expression was calculated using the formula 2^-△△Ct^. Solanum tuberosum translation elongation factor 1α gene (*StEF1α*) served as an internal control. The specific primers were listed in **Table 1**.

**Table 1:** Specific primers used in this study.

Gene ID	Gene	Forward (5’-3’)	Reverse (5’-3’)	Product length (bp)
XM_006347752.2	*StEf1α*	GGTTGTATCTCTTCCGATAAAGGC	GGTTGTATCTCTTCCGATAAAGGC	132
XM_015308529.1	*StP5CS*	TGCAATGCAATGGAAACGCT	ACAATTTCCACGGTGCAAGC	194
AY442179	*StCAT*	CCATGCTGAGGTGTATCCTATTC	CCTTTCTCCTGGTTGCTTGA	100
AF354748	*StSOD*	CATTGGAAGAGCTGTTGTTGTT	ATCCTTCCGCCAGCATTT	96
XM_006362636.2	*StPOD*	AGATGTTGTGGCCATGTCTGG	GCTTGTGTTGAAGGATGGAGC	118
PGSC0003DMT400006011	*StGATA1*	TGGAGATCGCAACTCCAGAAG	GCATTGCACAGCGACTTAGG	196
PGSC0003DMT400066953	*StGATA2*	CTCTGCGTTCCCAGTGATGA	TTCCGTGAAACGACGCAGTA	136
PGSC0003DMT400063771	*StGATA3*	TCCGACCCAAAAGGAGGAATC	TCCCACAAGCATTGCACAGT	137
PGSC0003DMT400061125	*StGATA4*	ATTGTGGCATCAAGCAAACCG	CTGTTTGCATGTCGGGGAAC	130
PGSC0003DMT400083773	*StGATA5*	CACCAAGATAGACACCAGCAAAC	TAGCGAATGTGAGGAGTAGGGTT	136
PGSC0003DMT400083774	*StGATA6*	GTGGAAGTGGAGGAGGAATAGAG	CCACACCATTCATCTCCATAGAAC	131
PGSC0003DMT400027729	*StGATA7*	CCCTGTAGATAGCGGCAGAGTTA	CCTCACACCCTCCTAATAATAGCA	142
PGSC0003DMT400027731	*StGATA8*	TGTGGGCTTATGTGGGCAAA	AGCTTCTTGCATATCCTCTTGAT	165
PGSC0003DMT400027733	*StGATA9*	CAGTTAACATTGATGAAGAGCAGGA	GCAGGCCAGTGAACACTCAT	130
PGSC0003DMT400027730	*StGATA10*	ACCACTGCTGAGCTGGATATG	TCCTGCTCTTCATCAATGTTAACTG	101
PGSC0003DMT400027728	*StGATA11*	ATGTGGGCAAACAAGGGTAT	CAGAAAGGAGAACTATCAGCAAAGT	187
PGSC0003DMT400008112	*StGATA12*	CTTCTTCTCCAACCTCTTCGTC	GGTCTCTGCTGATGGATTCTTT	142
PGSC0003DMT400008111	*StGATA13*	ACTCCCTATTCCGGTTGATGA	CGGTTCAGACCGGAACTCTG	145
PGSC0003DMT400031356	*StGATA14*	GAACTCAGTCTTCCTGGGGC	TCGTTCGGCACTAAACGGAA	192
PGSC0003DMT400040074	*StGATA15*	TTCTTCTTCGTCTTCCGTTGAT	ATGTAAGAAGAAGAAGAGCGACG	119
PGSC0003DMT400009117	*StGATA16*	AGTGAAATCAGTGTTCCGACTG	CCGGCAGGATAAGCAAGTGA	101
PGSC0003DMT400009118	*StGATA17*	CTTGTGGTGTTCGGTTCAAATC	TTTCTTCTGATTCCTTCTTCCG	137
PGSC0003DMT400009031	*StGATA18*	CATCGTTGTAGTGGGAGTATGGT	TGATAAGGCGAGTAGAAGGAGTTC	189
PGSC0003DMT400089018	*StGATA19*	CAAATTTCACCGTCTCTCTCACA	GAAGCTGTCCATCCCCTGC	100
PGSC0003DMT400006491	*StGATA20*	CGAACTCTGCGTTCCGTTTG	TGAACTGTCGGTGGTGATGG	148
PGSC0003DMT400070134	*StGATA21*	ATTCCTGACCTCAAGTCCTGTTT	AGAAACAGGACTTGAGGTCAGGA	124
PGSC0003DMT400070133	*StGATA22*	TTGGAACGATCCGTTGCCTG	AACGATATCCTCGTACGGAACT	106
PGSC0003DMT400070135	*StGATA23*	TCGGATTTCGTGGATGAGATAG	TCCTTACAATCAACAGCGTCAA	112
PGSC0003DMT400024208	*StGATA24*	TTGTGCTGATCTGGAAAAGAATC	TGCAGGAATGACGACCTCAG	154
PGSC0003DMT400024207	*StGATA25*	ATAGTGTCAGGAAAGAGGTTGCT	ACAGTTACAGAATGTTGTGTGCC	141
PGSC0003DMT400024206	*StGATA26*	GATGGAGGAGAAGAGACTATGGAT	TAGAAACTTCCACAACTCCACCT	123
PGSC0003DMT400024205	*StGATA27*	GGGAACTCCTGACAATCCCG	ACAGGCAGTCAACCTCAGTT	203
PGSC0003DMT400069864	*StGATA28*	AGCCCTTCATTTCCTGATTATGT	ACTGAATTTGGGCTGTGGTGA	137
PGSC0003DMT400069865	*StGATA29*	GCCCTTCATTTCCTGATTATGT	GTTGTTGTTGTTGTTGTTGCTG	172
PGSC0003DMT400060240	*StGATA30*	CAGCAGCAACAGTGAAGATAGTAA	AATGCTGCTTGTTCTACTTCTCC	167
PGSC0003DMT400060241	*StGATA31*	GGTGGATCTAAGTGATAAACAGGGT	GCAGGTCCACCTCTCCAAAG	141
PGSC0003DMT400060242	*StGATA32*	GCAGCAGCAACAGTGAAGATAGTA	ATGCTGCTTGTTCTACTTCTCCAA	167
PGSC0003DMT400059990	*StGATA33*	AGCTCTCAGTTCCGTATGAGG	GCCTTGGGATAACGGCTCTT	134
PGSC0003DMT400068348	*StGATA34*	ACAGCCTTCTCAAGGACACA	GCTTTCTTTGCACCTGCATACT	148
PGSC0003DMT400068347	*StGATA35*	GACATCCGAACTCAATAGGTAGAG	GTAGACAATCGTGAATAAGCCTCA	102
PGSC0003DMT400068346	*StGATA36*	AGTGACAAGCCTATGGTCTCTGTT	GGTAGACAATCGTGAATAAGCCTC	155
PGSC0003DMT400068349	*StGATA37*	GAAGTTACAGGAGGGCCCAA	CTGAAGCGAAATGGTCTGCAT	103
PGSC0003DMT400062488	*StGATA38*	CGAGGAAGATTGGGATGCGA	GGGACTCCAGAAATTCGTTAGGA	146
PGSC0003DMT400011449	*StGATA39*	TGGGAGATCAAAAGCAACAACC	CCACATGCGTTACACAATGACTTA	145
PGSC0003DMT400052800	*StGATA40*	TCCGAAGTGTTCAGGTGCAA	AGGCAAACGAGCTTCTTGGA	128
PGSC0003DMT400052799	*StGATA41*	AGAACCTTGTGACTTTGAGGAACA	GAGCCAGAACTTGACCTATTGCTA	188
PGSC0003DMT400052801	*StGATA42*	TCCGAAGTGTTCAGGTGCAAA	TTGGACTGAAGCAAGAGCCAT	113
PGSC0003DMT400074935	*StGATA43*	TTGTCCGGAAGCAATCACCC	CAGCCATATCTTCATATGGAACGG	100
PGSC0003DMT400067506	*StGATA44*	GTCGGTTGACAACAAGCACC	GGTGGTCCCTGCTCCTTTTA	170
PGSC0003DMT400067505	*StGATA45*	ACAACAATGCTCATACTTCTCTGG	GGCTTCTGATTCTTCTTCTCTACC	144
PGSC0003DMT400081417	*StGATA46*	CATCAGGTCCCAAGTCGTTG	GCCATCAATAATATCGCCGCT	187
PGSC0003DMT400030274	*StGATA47*	CAACTGCATGTTTCATGGTGGA	TTCTTCCTCTACACACTCAGGG	179
PGSC0003DMT400030276	*StGATA48*	GTGGATGATGACCTTCTCAACTTC	GAAGAAGGCTAACAAGAGGGTTTG	135
PGSC0003DMT400030708	*StGATA49*	ACCAACCACCTCCTACCGAT	TGCTACATCATCACTCGGAACA	110
PGSC0003DMT400020876	*StGATA50*	CATCCACACCCTCCGATCAA	CGAGGACGTACGGGAATGAC	152
PGSC0003DMT400020875	*StGATA51*	ACATCCACACCCTCCGATCA	ACTCTTTCCACCAGAGCAGG	112
PGSC0003DMT400000761	*StGATA52*	AGCAACAGCTCTTCCAACAAC	CATGCGTTACAAAGAGACTTAGGG	119
PGSC0003DMT400000760	*StGATA53*	CTCAAACTCTCACAGGAAAGTCGT	TACTCATAGGAACAAACTCTGGCG	172
PGSC0003DMT400000762	*StGATA54*	CTGATTACAGCAGCAACAGCTC	CCACATGCGTTACAAAGAGACTTA	133
PGSC0003DMT400011779	*StGATA55*	GACCTGCTGGACCTAAGTCAT	TTTCTCCGCTGCTGCTTGTA	186
PGSC0003DMT400011778	*StGATA56*	ATGTGGAATAAGGAGCAGGAAGA	GCTACTCTGGTTCTGAGGATGATG	120
PGSC0003DMT400011780	*StGATA57*	ACCTGCTGGACCTAAGTCATTGT	TGCTACTGCTATTGCTACTCTGGTT	164

The gene sequences described in this article are available at the National Center for Biotechnology Information (https://www.ncbi.nlm.nih.gov/) and Potato Genomics Resource (http://solanaceae.plantbiology.msu.edu/index.shtml) Heard the following login number.

### Subcellular localization

Protein-coding sequence of *StGATA2* was amplified and ligated to the expression vector pPBI121-EGFP, using the following primers: forward, 5’-CTCGAGATGGATGTCTACGGC GTGCACTCT-3’ and reverse, 5’-GTCGACGCAGACCCGAAAGTGATGTCCGTACATTC-3’. The constructed plasmid was transformed into *Agrobacterium tumefaciens* GV3101. The transformed strain infiltrated tobacco epidermal cells in accordance with a previous method reported by Sparkes et al. (2006). The green fluorescence was detected 48 h after infiltration under a Leica TCA confocal scanning laser microscopy (Leica, Weztlar, Germany).

### Phylogenetic analysis and amino acid alignment

Phylogenetic tress for GATA domain-containing homologs with highly similarity was constructed using MEGA 5.05 Software. Multiple-sequence alignment of GATA domain-containing amino acid sequence was analyzed using DNAman tool (Lynnon Biosoft, San Ramon, CA, USA)

### Measurements of plant growth

Potato plants were imaged 2 days after heat stress. Potato plants cultivated at 35°C were transferred to 22°C conditions and maintained for 7 days. CK plants were cultivated at 22°C for 2 days. The plants were measured for plant height, plant fresh weight (FW), plant dry weight (DW), root FW and root DW.

### Stomatal apertures

Stomatal apertures were examined according to a previous method reported by Tricker et al. (2005). After 35 days of cultivation, transgenic or non-transgenic plants were subjected to heat stress treatment for 24 h or 48 h. The comparable fully-expanded leaves of transgenic or non-transgenic plants were selected between 9:30-11:00 am for measuring stomatal aperture. Clear nail polish was applied to the third fully expanded fully functional leaf located at the top of the plant, followed by covering with scotch tape. The leaves were mounted on glass slides and imaged using a Zeiss-Axiolmager M2 microscope (Zeiss, Germany). Three biological replicates were performed for each condition separately.

### Net photosynthetic rate, transpiration rate, and stomatal conductance

The third leaf from the plant top was collected when it fully expands during 9:30-11:30. Net photosynthetic rate, transpiration rate and stomatal conductance were examined using a portable photosynthetic LI-6400XT system (Li-COR, Lincoln, NE, USA). The photon flux density was set as 1,500 μmol·m^-2^·s^-1^. The relative humidity in leaf chamber was 50%-70%. CO_2_ concentration was 400 μmol/mol.

### Contents of H_2_O_2_, proline, and MDA, and activities of CAT, SOD, and POD

To assess physiological and biochemical changes in plants under heat stress, 5-week-old *StGATA2* transgenic plants and non-transgenic control plants grown under greenhouse conditions were subjected to heat stress treatments at 30°C and 35°C. Contents of H_2_O_2_, proline, and malondialdehyde (MDA) and activities of catalase (CAT), superoxide dismutase (SOD), and peroxidase (POD) were determined according to our previous methods (Zhu et al., 2021).

### Statistical analysis

All experiments were carried out with three biological replicates and three technological replicates. Data are shown as the mean ± standard deviation. Statistical analysis was done with IBM SPSS 19.0 Statistical Software (IBM, Chicago, IL, USA) and GraphPad Prism Software (GraphPad, San Diego, CA, USA). Multiple comparisons were analyzed by one-way ANOVA with Tukey test or Dunnett’s T3 for *post-hoc* analysis or two-way ANOVA corrected by Sidak’s multiple comparisons test. Histograms were computed with GraphPad Prism software, heatmap by the pheatmap function in R version 4.2.3.

## Results

### Expression signatures of *GATA* family genes in potato plants in response to heat stress

**Figure 1:**
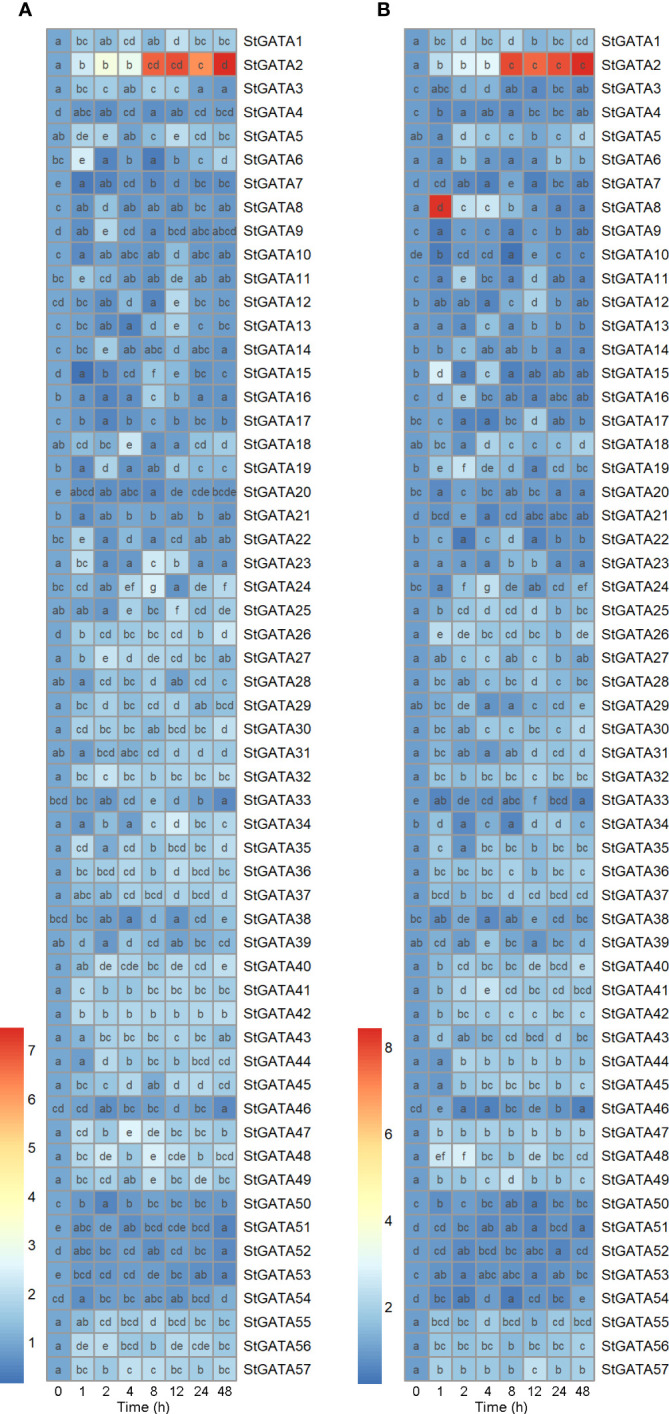
Heatmap suggesting mRNA expression of *StGATA* family genes in potato leaves in response to heat stress. Five-week-old normally grown plants were subjected to 0 h, 1 h, 2 h, 4 h, 8 h, 12 h, 24 h, or 48 h of **(A)** mild (30°C) or **(B)** acute (35 °C) heat stresses. Multiple comparisons were analyzed by one-way ANOVA with Tukey test or Dunnett’s T3 for *post-hoc* analysis. Difference letters indicates the difference between the two groups (p<0.05).

To provide an overview of expression patterns of *GATA* family genes, we cultivated potato plants under heat stresses. qRT-PCR results showed that mRNA expression of the 57 members from *GATA* family genes were distinctly altered during mild (30°C) or acute (35°C) heat stresses (0-48 h), which was presented in heatmaps (**Figures 1A, B**). Importantly, under heat stress conditions, *StGATA2* expression was induced 1-48 h after heat stress (30°C and 35°C) treatment and peaked at 48 h with 7.5-fold and 8.4-fold increases in mRNA expression, respectively (**Figure 1**). Notably, *StGATA2* transcript levels were maintained at higher levels (expression increased more than 7 folds) at 8 h, 12 h, 24 h and 48 h after heat stress treatment (P < 0.05). The results indicated that heat stress at 30°C and 35°C induced a sustained and stable high expression of *StGATA2*. Consequently, we speculated that *StGATA2* gene may play molecular functions in response to heat stresses.

### Potato plants encoding GATA2 gene with CTHC-X18-CNAC motif

**Figure 2:**
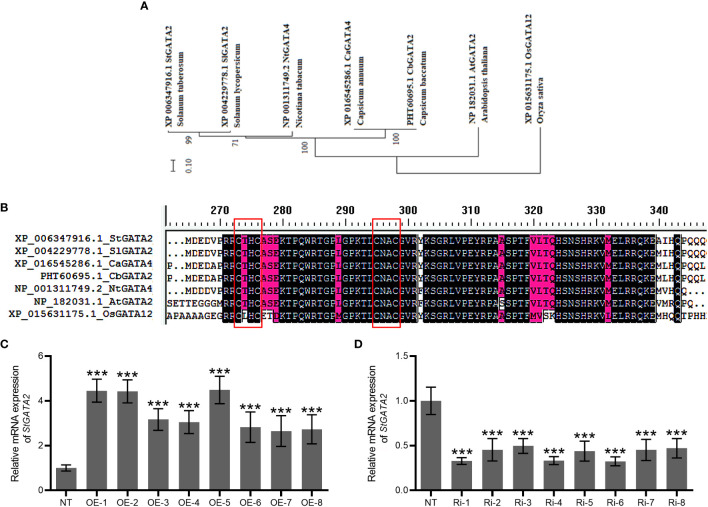
Bioinformatic analysis of StGATA2 protein and construction of *StGATA2* overexpression or under-expression plants. **(A)** Phylogenetic tree and schematic representation of GATA factors from *Solanum tuberosum* (St), *Solanum lycopersicum* (Sl), *Nicotiana tabacum* (Nt), *Capsicum annuum* (Ca), *Capsicum baccatum* (Cb), *Arabidopsis thaliana* (At), *Oryza sativa* (Os). The scale bar represents the difference degree of amino acid sequences. **(B)** Multiple-sequence alignment of conserved single CTHC-X18-CNAC motif highlighted in red boxes. **(C, D)** The quantification of *StGATA2* mRNA expression in potato plants; NT, non-transgenic plants; OE, pBI121-EGFP-StGATA2-transgenic lines; Ri, pART-StGATA2-RNAi-transgenic plants; Mean ± standard deviation; Student’s t-test, ***p<0.001 (n = 9).

**Figure 3:**
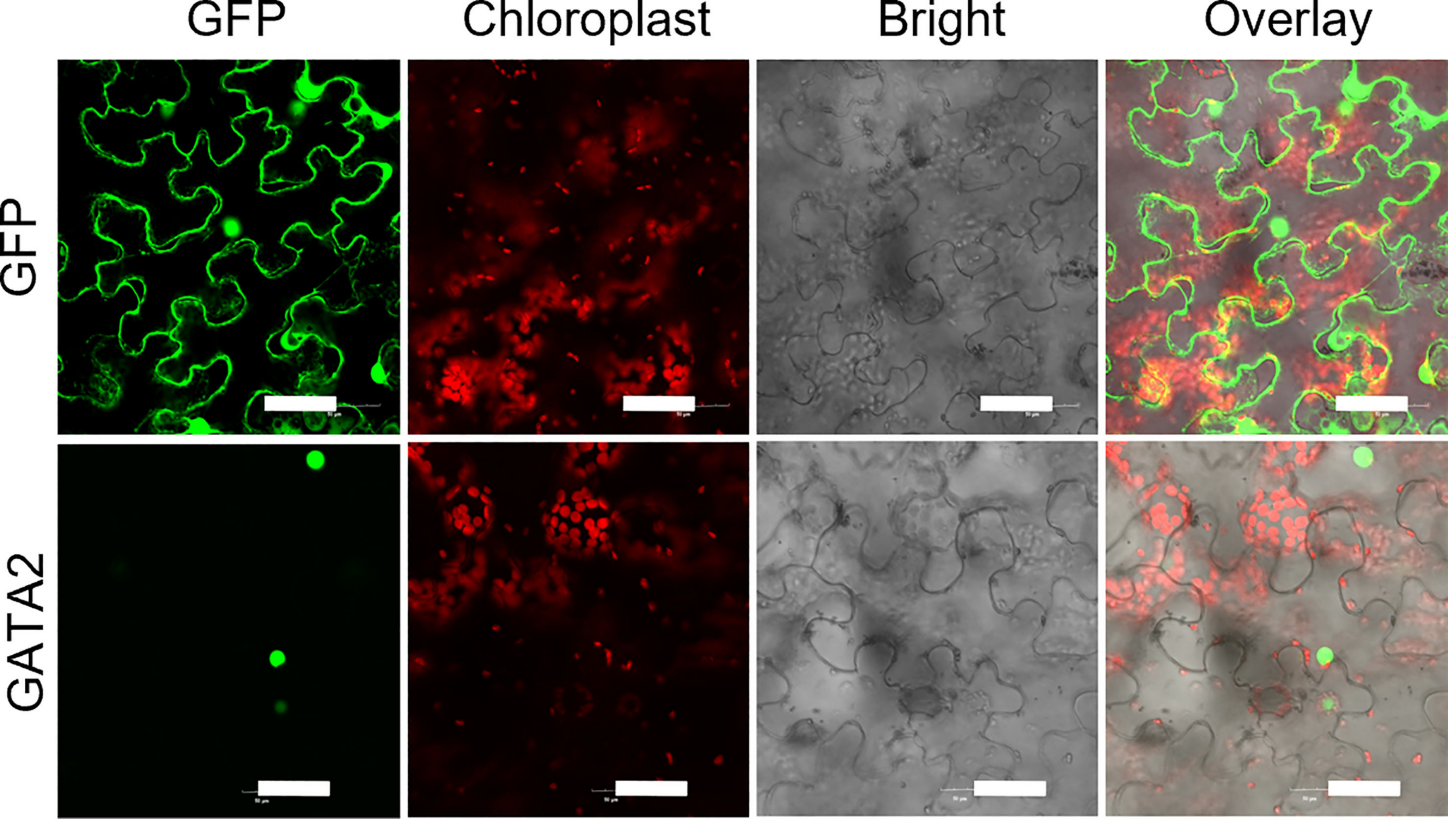
StGATA2 protein locates on the cellular nucleus of tobacco epidermal cells. GFP-StGATA2 fusion protein was transiently expressed in tobacco leaves and observed using a laser scanning confocal microscope. Bar = 50 μm.

Phylogenetic tree of GATA factors was generated, including *Solanum tuberosum* GATA2 (StGATA2), *Solanum lycopersicum* GATA2 (SlGATA2), *Nicotiana tabacum* GATA4 (NtGATA4), *Capsicum annuum* GATA4 (CaGATA4), *Capsicum baccatum* GATA2 (CbGATA2), *Arabidopsis thaliana* GATA2 (AtGATA2), *Oryza sativa* GATA12 (OsGATA12) (**Figure 2A**). *Solanum tuberosum* GATA2 contains CTHC-X18-CNAC motif (**Figure 2B**). To examine *StGATA2* function, we obtained *StGATA2* overexpression plants by expressing pBI121-EGFP-StGATA2 and generated a loss-of-function plants by introducing pART-StGATA2-RNAi. The relative mRNA expression of *StGATA2* in transgenic plants were shown in **Figures 2C, D**, which significantly increased or knocked down when compared to the wild type (p<0.001). Amongst the overexpression lines, OE-1, OE-2 and OE-5 were selected as the significant over-expressors, and Ri-1, Ri-4 and Ri-6 as the significant under-expressors used for the functional analysis in response to heat stresses. StGATA2 protein obviously located in the nucleus (**Figure 3**). The subcellular localization of GATA2 to the nucleus is the structural premise for DNA binding and transcription activation.

### Effects of *StGATA2* on plant morphological phenotypes and growth in response to heat stresses

**Figure 4:**
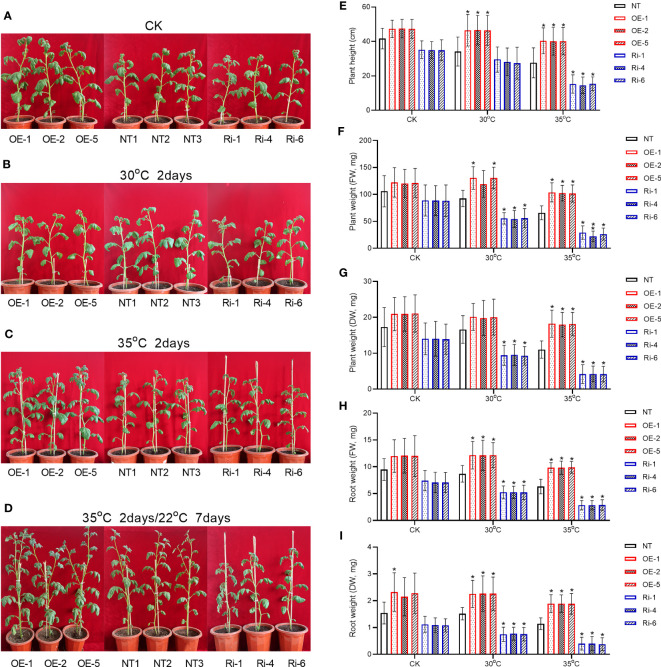
Growth characterizations of *StGATA2* over-expression or under-expression plants in response to heat stresses. **(A–D)** Representative photographs of potato plants with the specified genotypes (NT, OE, and Ri); Five-week-old plants were cultivated under **(A)** 22°C (CK), **(B)** 30°C (30°C), **(C)** 35°C, or **(D)** 35°C followed by moving to 22°C condition; Plants in A, B, and C were imaged 2 days after heat stress; After heat stress, plants in D were imaged 7 days after normal cultivation. **(E)** Plant height, **(F)** plant fresh weight (FW), **(G)** plant dry weight (DW), **(H)** root FW, and **(I)** root DW of transgenic or non-transgenic plants were measured 2 days after cultivation under the specified conditions (22°C, 30°C, and 35°C). NT, non-transgenic plants; OE, pBI121-EGFP-StGATA2-transgenic lines; Ri, pART-StGATA2-RNAi-transgenic plants; Mean ± standard deviation; Ordinary two-way ANOVA with Tukey’s multiple comparisons test, *p<0.05 (n = 9).

Next, we cultivated the 5-week-old transgenic or non-transgenic plants under heat stresses for 2 days, and investigated whether *StGATA2* gene is involved in regulating plant morphology or growth such as plant height, plant weight and root weight. The morphological features of the plants were imaged 2 days after cultivation at 22°C (CK), 30°C, or 35°C. **Figure 4A** showed that neither *StGATA2* over-expression nor under-expression affected plant morphological structure, compared to wild-type plants at 22°C. However, the height of OE plants in **Figure 4B** or Ri plants in **Figure 4C** was significantly different from the non-transgenic plants.

The statistical analysis results revealed that under mild heat stress (30°C), OE plants were characterized by increased plant height (**Figure 4E**), while plant fresh weight (**Figure 4F**), plant dry weight (**Figure 4G**), root fresh weight (**Figure 4H**) or root dry weight (**Figure 4I**) were unchanged (p>0.05) relative to NT plants (p<0.05). In contrast, the height of Ri plants was not significantly different from NT plants (p>0.05) (**Figure 4E**), while *StMAPK1* under-expression reduced plant fresh weight (**Figure 4F**), plant dry weight (**Figure 4G**), root fresh weight (**Figure 4H**) or root dry weight (**Figure 4I**) (p<0.05) 2 days after growth at 30°C. As for the acute heat stress (35°C), OE plants displayed significant increases in plant height, plant fresh weight, plant dry weight, root fresh weight, and root dry weight, which were significantly decreased in Ri plants compared to non-transgenic plants (p<0.05).

Further, we compared the morphological structures of transgenic plants to non-transgenic plants 7 days after the plants receiving heat stresses were cultivated at 22°C. **Figure 4D** showed that compared to the non-transgenic plants, OE plants showed an increased plant height and more apical branches. However, Ri plants grows slowly, with a shorter height compared to NT plants. These results indicated that *StGATA2* is implicated in the growth of potato plants in response to heat stresses, especially acute heat stress.

### *StGATA2* over-expression enhanced photosynthetic rate and inhibited transpiration in response to heat stresses

**Figure 5:**
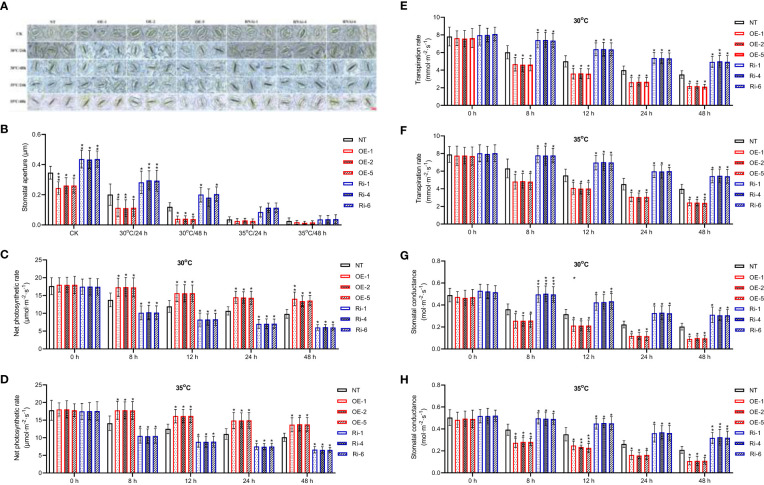
Stomatal apertures and photosynthesis of *StGATA2* over-expression or under-expression plants in response to heat stresses. **(A)** Representative images of *Solanum tuberosum* stomata; The scale bar equates to 10 μm. **(B)** Stomatal apertures depicted by the ratio of pore width to length. **(C, D)** Net photosynthetic rate, **(E, F)** transpiration rate and **(G, H)** stomatal conductance of potato leaves. The plants were measured 2 days after cultivation under the specified conditions (22°C, 30°C, and 35°C). NT, non-transgenic plants; OE, pBI121-EGFP-StGATA2-transgenic lines; Ri, pART-StGATA2-RNAi-transgenic plants. Mean ± standard deviation. Ordinary two-way ANOVA with Tukey’s multiple comparisons test, *p<0.05, **p<0.01,***p<0.001 (n = 9).

Measurements revealed that average ratio of stomal width to length was significantly decreased in *StGATA2* overexpressing plants compared with NT plants, 24 or 48 h after mild heat stresses (p<0.05, p<0.01) (**Figures 5A, B**). *StGATA2* under-expression resulted in a significant increase in stomatal aperture (p<0.05), 24 h or 48 h after mild or acute heat stresses. Under heat stress conditions, the net photosynthetic rate was increasingly enhanced in OE plants, while decreased in Ri plants (p<0.05) (**Figures 5C, D**). Additionally, there were evidence of *StGATA2* overexpression inhibiting transpiration rate (**Figures 5E, F**) and decreasing stomatal conductance (**Figures 5G, H**) (p<0.05, p<0.01). Comparing to the NT plants, 30°C or 35°C heat stresses resulted in significant increases in transpiration rate and stomatal conductance of *StGATA2* under-expression plants (p<0.05, p<0.01, p<0.001). All of these results confirmed that *StGATA2* is closely correlated with heat stress-induced modifications in photosynthesis and transpiration rate.

### Biochemical and genetic responses of *StGATA2*-transgenic potato plants to heat stress

**Figure 6:**
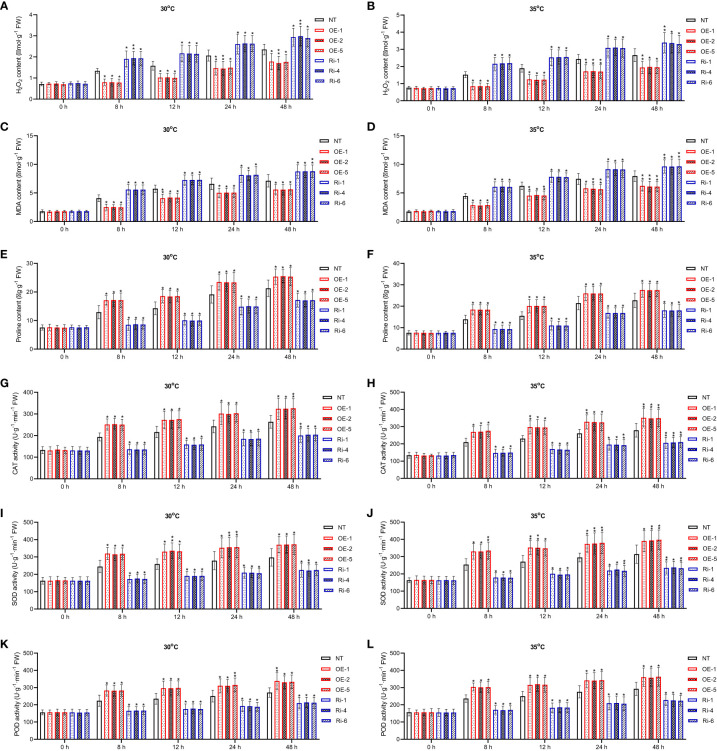
Effects of *StGATA2* expression on biochemical indexes of potato plants in response to heat stresses. **(A, B)** H_2_O_2_ content, **(C, D)** MDA content, **(E, F)** proline content, **(G, H)** CAT activity, **(I, J)** SOD activity, and **(K, L)** POD activity in potato leaves were estimated 2 days after cultivation under the specified conditions (22°C, 30°C, and 35°C). NT, non-transgenic plants; OE, pBI121-EGFP-StGATA2-transgenic lines; Ri, pART-StGATA2-RNAi-transgenic plants. Mean ± standard deviation. Ordinary two-way ANOVA with Tukey’s multiple comparisons test, *p<0.05, **p<0.01, ***p<0.001 (n = 9).

Biochemical changes in potato plants were assayed to evaluate the capacity of *StGATA2* to trigger protective mechanisms against heat stress. There is a significantly increase in contents of H_2_O_2_, MDA, and proline, as well as the activity of CAT, SOD and POD, following mild and acute heat stress treatment (p<0.05, p<0.01) (**Figure 6**). Compared to the NT plants, *StGATA2* overexpression significantly restrained the accumulation of H_2_O_2_ and MDA, while further induced the generation of proline (**Figures 6A–F**). Under heat stress conditions, *StGATA2* overexpression enhanced the activity of CAT, SOD and POD compared to the non-transgenic plants (**Figures 6G–L**). However, there was an opposite trend toward the contents of H_2_O_2_, MDA and proline, as well as the activity of CAT, SOD, and POD in the Ri plants following heat stress, compared with OE plants. These data suggested that *StGATA2* overexpression induced biochemical responses of potato plant against heat stress.

**Figure 7:**
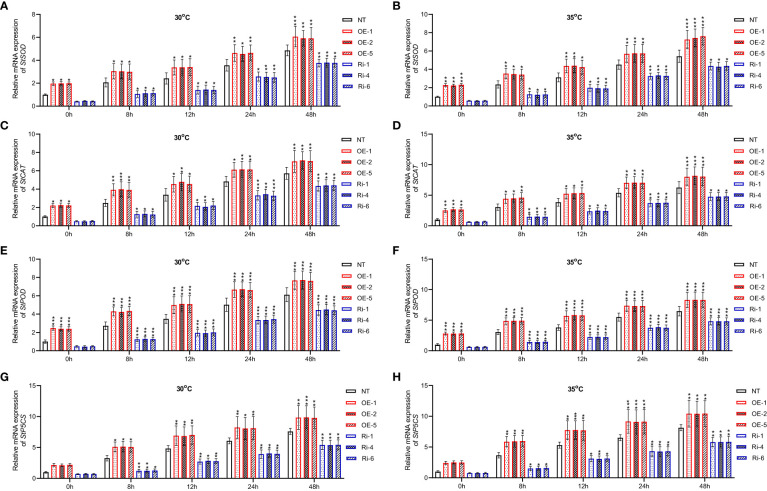
Changes in relative mRNA expression of heat-stress responsive genes in *StGATA2* over-expression or under-expression plants in response to heat stresses. **(A, B)** mRNA expression of *StSOD*, **(C, D)** *StCAT*, **(E, F)** *StPOD*, and **(G, H)** *StP5CS* in potato leaves were estimated 2 days after cultivation under the specified conditions (22°C, 30°C, and 35°C). NT, non-transgenic plants; OE, pBI121-EGFP-StGATA2-transgenic lines; Ri, pART-StGATA2-RNAi-transgenic plants. Mean ± standard deviation. Ordinary two-way ANOVA with Tukey’s multiple comparisons test, *p<0.05, **p<0.01, ***p<0.001 (n = 9).

Besides, under heat stress conditions, mRNA expression of heat stress responsive genes was increased in non-transgenic, including *StSOD* (**Figures 7A, B**), *StCAT* (**Figures 7C, D**), *StPOD* (**Figures 7E, F**), *StP5CS* (**Figures 7G, H**) (p<0.05, p<0.01, p<0.001). However, *StGATA2* over-expression further elevated the transcription of these 4 heat stress-responsive genes following heat stress treatment, compared to non-transgenic plants. In contrast, Ri plants showed the opposite results. This analysis indicated that *StGATA2* overexpression induced genetic responses of potato plant against heat stress.

## Discussion

High temperature limited plant morphophysiological growth, photosynthesis, dark respiration, carbohydrate metabolism (Lafta and Lorenzen, 1995; Fahad et al., 2016), root system architecture (Luo et al., 2020), stomal regulation and water use (Körner, 2006). *GATA* family genes are heat-responsive genes, which has been identified in cucumber (Zhang et al., 2021), wheat (Feng et al., 2022b), and pepper (Yu et al., 2021). However, these studies only characterized the expression pattern of the *GATA* family genes under heat stress and lacked validation of its function. In the present study, we present the first evidence that *StGATA2* plays an important role in maintaining plant growth against heat stresses, through the regulation of heat-responsive gene expression in *Solanum tuberosum*.

Genome-wide analysis has identified the *GATA* family genes in wheat (Feng et al., 2022b), pepper (Yu et al., 2021), cucumber (Zhang et al., 2021), rice (Gupta et al., 2017), and foxtail millet (Lai et al., 2022). Based on the whole potato genome data, Yu et al. systematically identified 49 GATA proteins, of which mRNA expression is affected by *Ralstonia solanacearum*, abscisic acid and methyl jasmonate (Yu et al., 2022). Our previous study identified 57 GATA proteins randomly distributed on 12 chromosomes (unpublished data). The expression profile of *GATA* genes was significantly altered when plants are subjected to heat stress (Yu et al., 2021; Feng et al., 2022b), suggesting their potential roles involved in biological functions associated with heat tolerance. The expression pattern of *GATA* genes is still unexplored when potato plants are exposed to heat stresses. As a result, we cultivated potato plants under mild and acute heat stresses and determined mRNA expression of *GATA* genes. In mature potato plants, we noted an evident alteration in mRNA expression of *GATA* family genes under heat stress conditions during the observed intervals. We found a substantially and stably up-regulated gene *StGATA2* after heat stress treatment. This suggested that *StGATA2* is a positive heat stress-responsive gene in the wild-type potato plant. *StGATA2* gene was then selected for constructing transgenic plants to identify its roles in response to heat stress.

StGATA2 belongs to 18-residue loops (i.e. CX_2_CX_18_CX_2_C; also called zinger finger type IVb). The zinc finger motifs are categorized into two groups: those with 17-residue loops (CX_2_CX_17_CX_2_C zinc finger type IVa) and those with 18-residue loops (CX_2_CX_18_CX_2_C zinc finger type IVb), which are based on the spacing between the cysteine pairs at the zinc finger loop (Park et al., 2006). GATA transcription factors play a crucial role in plant growth, biomass accumulation and plant height, of which *PdGNC* have been confirmed in poplar, for example (An et al., 2019). Studies have revealed the involvement of *GATAs* in responding to multiple stresses when the crops are exposed to salinity, drought, exogenous ABA, acid, alkali, dark, flooding, heat, and cold (Gupta et al., 2017; Yu et al., 2021; Zhang et al., 2021; Feng et al., 2022b; Lai et al., 2022). For instance, in tomato plants, *SlGATA17* expression was induced by heat stress (Wang et al., 2023). Transgenic Arabidopsis plants overexpressing GATA factors ZIM show enhanced petiole and hypocotyl cell elongation (Shikata et al., 2004). However, limited studies have been conducted to demonstrate the roles of *GATAs* in plant growth in response to heat stress. Potato is characterized by specific temperature requirements and develops best at about 20°C. Subsequently we constructed the loss-of-function and gain-of-function variants to examine the role of *StGATA2* in potato plant growth. Our results confirmed that potato plant growth was greatly reduced at 30 °C and 35°C higher than optimum, responding with decreases in plant height and weight. The transfer of plants from 35°C to 22°C had apparent effect on shoot growth. Besides, He et al. have validated that *GATA* is a crucial regulator of the flag leaf development in rice (He et al., 2018). These results suggested that *StGATA2*, a member of the *StGATA* family genes, is involved in the growth process of several organs such as root, shoot and leaf of the potato plants.

Stomata protects plants against immediate or long-term injuries caused by environmental alterations. It was recently shown that heat stress initiated a rapid local and systemic stomatal opening responses (Devireddy et al., 2020). In Arabidopsis, Klermund et al. reported that LLM-domain B-class *GATA* genes strongly promotes stomata formation, most strikingly in hypocotyls but also in cotyledons (Klermund et al., 2016). Whereas it is still unclear about how *GATA* genes affect the stomatal aperture. In our study, consistent heat stress decreases the stomatal aperture, and *StGATA2* expression significantly further inhibited the stomatal aperture, which may play roles in leaf temperature, CO_2_ exchange rates and water vapor loss. In poplar, *PdGNC* affects chlorophyll content and photosynthetic rate (An et al., 2019). The conserved GATA transcription factor mediates plant architecture and chloroplast development by altering chlorophyll, chloroplast number, photosynthesis, and amnio acid and starch biosynthesis, which may be associate with a semidwarf phenotype of the transgenic rice (Hudson et al., 2013). Transcriptomic analysis found that *PdGNC* may potentially mediate nitrogen uptake in the root, cell division and carbohydrate utilization in the stem, and photosynthetic electron transfer and carbon assimilation in the leaf (An et al., 2019). However, the biological function of *GATAs* has not been studied under environmental stresses. Our data clearly demonstrated that *StGATA2* was involved in maintaining photosynthetic rate and mitigating respiratory rate.

Catalase functions as antioxidant enzyme, dissolving hydrogen peroxide into water and oxygen peroxisomes, of which encoding genes were found with cis-elements related to stress responses and plant hormones signaling in potato plants (Jbir Koubaa et al., 2023). Superoxide dismutase encoded by *StCuZnSODs*, *StFeSOD3* and *StMnSOD* play a significant role in catalyzing the conversion or dismutation of toxic superoxide anion radical into H_2_O_2_ and O_2_, representing the first line of antioxidant defense against ROS induced by heat stress (Rudić et al., 2022). Peroxidase also serves as ROS-scavenging enzyme, catalyzing the conversion of H_2_O_2_ into H_2_O with ascorbate as a specific electron donor (Caverzan et al., 2012). The expression of peroxidase-encoding gene is regulated in response to abiotic stresses such as heat stress during plant development (Caverzan et al., 2012). Our data suggested that the changes in enzyme activities of SOD, CAT, and POD at high temperature were coordinated with alterations in the transcription of the protein-encoding genes. The increases in activities of antioxidant enzymes at high temperature is more likely due to an increase in *StGATA2* expression. In the present experiments, increased proline content was observed at high temperature, which has been related to elevated transcripts of *StP5CS*. A study has confirmed the efficacy of proline in countering the damages in plant growth and enzymes of carbon and antioxidative metabolism (Kaushal et al., 2011). We found that *StGATA2* gene was associated with the maintained expression of *StP5CS* and proline level.

Plant GATAs containing GATA-type zinc fingers with two CX_2_C motifs interspaced by a 17-20 amino acid long loop recognize the DNA sequence W-G-A-T-A-R through a single type IV zinc finger (Patient and McGhee, 2002; Reyes et al., 2004). Sugimoto et al. reported that the GATA-type zinc finger region is required for binding activity and activate expression from *NtMyb2* promoter (Sugimoto et al., 2003). In reverse, Gupta et al. presented that the alternative splice variant of *OsGATA23* (*OsGATA23b*) was unable to respond to abiotic tresses, which throws a novel light on the tight regulation of the spliced variants of *OsGATA* genes in response to salinity, drought and exogenous ABA (Gupta et al., 2017). Wang et al. performed yeast one assay and dual-luc assay and confirmed that the GATA-box of *SlGATA17* promoter was bound by SlHY5 and its expression was then regulated, which causes the hypersensitivity of Arabidopsis to NaCl at the post-germination stage (Wang et al., 2023). However, it is not yet possible to explain how the StGATA2 gene regulates the expression of heat responsive genes based on the available results of this study. *GATA*-encoded proteins contain higher conserved DNA binding domain that consist of two zinc finger motifs and two adjacent stretches of basic amino acids (Molkentin, 2000). The N-terminal finger can interact with adjacent GATA DNA sequence elements or with protein cofactors (Trainor et al., 1996; Weiss et al., 1997). The nuclear localization and transcriptional activation domains of *GATA2*-encoding protein may promote or inhibit the transcriptional activation of *StSOD*, *StCAT*, *StPOD*, and *StP5CS*. However, the identification of the protein domain needs protein domain deletion analysis.

## Data availability statement

The original contributions presented in the study are included in the article/supplementary material. Further inquiries can be directed to the corresponding authors. The GATA protein sequences described in this article are available at the National Center for Biotechnology Information (https://www.ncbi.nlm.nih.gov/) with the following number: SlGATA2 (XP_004229778.1), StGATA2 (XP_006347916.1), CaGATA4 (XP 016545286.1), CbGATA2 (PHT60695.1), NtGATA4 (NP_001311749.2), AtGATA2 (NP_182031.1), OsGATA12 (XP_015631175.1).

## Author contributions

XZ, HD, and YZ planned and designed the research. XZ, HD, HJ, SC, ZC, SS, JT, and YZ collected the data. XZ, HD, HJ, SC, ZC, SS, JT, and YZ analyzed the data. XZ, HD, and YZ drafted the manuscript. All authors contributed to the article and approved the submitted version.
